# Editorial: Unraveling inflammatory pathways in sickle cell disease: molecular, cellular and translational insights

**DOI:** 10.3389/fimmu.2026.1796628

**Published:** 2026-02-06

**Authors:** Nicola Conran, Subarna Chakravorty, Renata Sesti-Costa

**Affiliations:** 1 Hematology Center, University of Campinas-UNICAMP, Campinas, São Paulo, Brazil; 2 Department of Paediatric Haematology, King’s College Hospital, London, United Kingdom

**Keywords:** endothelial cells, inflammation, leukocytes, pathophysiology, sickle cell anemia, sickle cell disease

Since its identification as a “molecular disease” by Linus Pauling and colleagues in 1949 (1), the genetic and molecular foundations of sickle cell disease (SCD) have fascinated the research community. While the disease’s underlying point mutation in the β-globin gene alters the structure and properties of hemoglobin, triggering erythrocyte sickling and hemolysis, we no longer view SCD strictly as a red blood cell (RBC) disorder (2). Current research now emphasizes coordinated interactions among multiple cell types and recognizes roles for innate immune pathways and inflammatory signaling in the disease (3, 4). These pathways shape the cellular responses that drive vaso-occlusive processes and trigger progressive organ damage and the diverse complications of the disease.

Leukocytes, particularly neutrophils and monocytes, are key to SCD pathophysiology. High leukocyte counts can predict disease severity (5, 6), and murine model studies have demonstrated a physical role for these cells in orchestrating the vaso-occlusive process by adhering to the vascular wall and capturing circulating RBCs, leading to blood vessel obstruction (7, 8). Beyond this mechanical disruption of microvascular hemodynamics, these cells function as primary inflammatory effectors that respond to disease-generated stimuli. One important focus of emerging research is neutrophil plasticity, where inflammatory signaling can influence the functional profile of these cells. Beyond neutrophil priming and activation in SCD, the accumulation of aged neutrophils (9) and low-density neutrophils (LDNs) (10) suggests functional shifts that may amplify disease pathology.

Gaartman et al., in their contribution to this Research Topic, explored this plasticity by comparing the phenotype and function of neutrophils in groups of SCD patients to those of ethnicity-matched and non-matched controls. Their study confirmed significant inter-individual variation in neutrophil phenotypes, in association with limited functional changes. Notably, the authors highlighted the importance of using ethnically-matched controls to investigate neutrophil phenotype in SCD, a disease that is associated predominantly with African and African descendant populations.

Expanding on this theme, Terrigno et al. compared neutrophil phenotypic and inflammatory profiles in SCD patients receiving “gold standard” therapies, namely hydroxyurea and chronic transfusion (CT). Supporting the known anti-inflammatory effects of hydroxyurea, lower neutrophil counts and numbers of aged neutrophils were identified in those taking this drug compared to the transfused group. This focus on aged neutrophils is reinforced by Gaartman et al. who detected a shift towards a seemingly “*younger*” neutrophil profile in patients during vaso-occlusive crisis. These studies suggest that while hydroxyurea may reduce the aged neutrophil population during steady state, the crisis state may accelerate myeloid egression (as indicated by increased neutrophil counts) and the sequestration of the aged neutrophil population in the vasculature. Terrigno et al. also reported that hydroxyurea therapy was associated with lower levels of several circulating chemokines, compared to CT, suggesting distinct inflammatory skews between the two treatments. Authors conclude that combining hydroxyurea with CT may be warranted to ensure improved dampening of the systemic inflammatory state in patients on regular CT, especially given the high clinical severity that typically prompts the initiation of transfusion.

Borges et al. demonstrated altered phenotype and function of circulating monocytes, another important cellular mediator of SCD pathophysiology (11). By comparing monocytes derived from diseased patients and healthy controls they show that circulating monocytes undergo sustained functional adaptation in response to repeated contact with sickled erythrocytes. This exposure promotes a coordinated shift in surface receptor expression that favors erythroid engagement, characterized by increased VCAM-1–mediated adhesion and attenuation of inhibitory signaling through reduced SIRP-α. Together, these changes lower the threshold for RBC internalization. Following erythrophagocytosis, monocytes activate conserved heme-handling pathways, including induction of heme oxygenase-1 and upregulation of ferroportin, enabling efficient degradation of hemoglobin-derived heme and subsequent iron export. The concurrent increase in CD206 expression suggests alignment with macrophage programs specialized for erythroid support and iron recycling. Through these interconnected mechanisms, circulating monocytes emerge as active regulators of RBC clearance and iron flux, linking erythrophagocytosis to inflammatory modulation and vascular dysfunction in SCD.

Beyond the focus on leukocytes in SCD, endothelial activation and injury play a central role in disease pathophysiology. Recurrent ischemia–reperfusion events, together with the sustained release of damage-associated molecular patterns (DAMPs) and inflammatory mediators, promote endothelial dysfunction and detachment of cells from the vessel wall, leading to elevated levels of circulating endothelial cells (CECs) in SCD (12, 13). In parallel, the chronic inflammatory milieu stimulates multiple cell types to release extracellular vesicles into the bloodstream, which can interact with and activate the endothelium, thereby contributing to vascular dysfunction and vaso-occlusive events (14, 15).

In this Research Topic, Beckman et al. rigorously validated pre-analytical and analytical methodologies for the quantification of CECs and endothelial extracellular vesicles (EEVs) in peripheral blood. Their study confirms and extends previous observations by demonstrating increased levels of both CECs and EEVs in SCD patients. Importantly, a detailed phenotypic analysis revealed that mature and resting CECs are the predominant subsets elevated in patients, whereas circulating EEVs largely originate from activated endothelial cells, as indicated by CD106 expression. Notably, the authors further demonstrate that, despite the marked elevation of soluble adhesion molecules and CECs in the plasma of SCD patients, these parameters do not reliably discriminate vaso-occlusive crises. In contrast, both total EEVs and activated EEVs were significantly increased during pain crises, highlighting their potential clinical relevance and role as rapidly signaling molecules. Although longitudinal studies are still required, these findings position endothelial extracellular vesicles as promising biomarkers for monitoring vascular dysfunction and therapeutic responses during vaso-occlusive episodes in SCD.

Collectively, this Research Topic underscores that a comprehensive understanding of the multicellular landscape and the specific contributions of distinct cell types in the pathophysiology of SCD is critical for improving clinical management, monitoring and patient stratification. The differential skewing of inflammatory profiles, signaling pathways and functional cell phenotypes during therapies may serve to guide the development of more targeted therapeutic strategies aimed at reducing complications associated with vaso-occlusion and the wide-ranging manifestations that are driven by inflammatory processes in this neglected and debilitating disease.
